# Artificial intelligence in hospital infection prevention: an integrative review

**DOI:** 10.3389/fpubh.2025.1547450

**Published:** 2025-04-02

**Authors:** Rabie Adel El Arab, Zainab Almoosa, May Alkhunaizi, Fuad H. Abuadas, Joel Somerville

**Affiliations:** ^1^Almoosa College of Health Sciences, Al Mubarraz, Saudi Arabia; ^2^Department of Infectious Disease, Almoosa Specialist Hospital, Al Mubarraz, Saudi Arabia; ^3^Department of Pediatric, Almoosa Specialist Hospital, Al Mubarraz, Saudi Arabia; ^4^Department of Community Health Nursing, College of Nursing, Jouf University, Sakaka, Saudi Arabia; ^5^Inverness College, University of the Highlands and Island, Inverness, United Kingdom; ^6^Glasgow Caledonian University, Glasgow, United Kingdom

**Keywords:** hospital-acquired infections, artificial intelligence, infection prevention, infection control, predictive analytics, infection surveillance, explainable AI

## Abstract

**Background:**

Hospital-acquired infections (HAIs) represent a persistent challenge in healthcare, contributing to substantial morbidity, mortality, and economic burden. Artificial intelligence (AI) offers promising potential for improving HAIs prevention through advanced predictive capabilities.

**Objective:**

To evaluate the effectiveness, usability, and challenges of AI models in preventing, detecting, and managing HAIs.

**Methods:**

This integrative review synthesized findings from 42 studies, guided by the SPIDER framework for inclusion criteria. We assessed the quality of included studies by applying the TRIPOD checklist to individual predictive studies and the AMSTAR 2 tool for reviews.

**Results:**

AI models demonstrated high predictive accuracy for the detection, surveillance, and prevention of multiple HAIs, with models for surgical site infections and urinary tract infections frequently achieving area-under-the-curve (AUC) scores exceeding 0.80, indicating strong reliability. Comparative data suggest that while both machine learning and deep learning approaches perform well, some deep learning models may offer slight advantages in complex data environments. Advanced algorithms, including neural networks, decision trees, and random forests, significantly improved detection rates when integrated with EHRs, enabling real-time surveillance and timely interventions. In resource-constrained settings, non-real-time AI models utilizing historical EHR data showed considerable scalability, facilitating broader implementation in infection surveillance and control. AI-supported surveillance systems outperformed traditional methods in accurately identifying infection rates and enhancing compliance with hand hygiene protocols. Furthermore, Explainable AI (XAI) frameworks and interpretability tools such as Shapley additive explanations (SHAP) values increased clinician trust and facilitated actionable insights. AI also played a pivotal role in antimicrobial stewardship by predicting the emergence of multidrug-resistant organisms and guiding optimal antibiotic usage, thereby reducing reliance on second-line treatments. However, challenges including the need for comprehensive clinician training, high integration costs, and ensuring compatibility with existing workflows were identified as barriers to widespread adoption.

**Discussion:**

The integration of AI in HAI prevention and management represents a potentially transformative shift in enhancing predictive capabilities and supporting effective infection control measures. Successful implementation necessitates standardized validation protocols, transparent data reporting, and the development of user-friendly interfaces to ensure seamless adoption by healthcare professionals. Variability in data sources and model validations across studies underscores the necessity for multicenter collaborations and external validations to ensure consistent performance across diverse healthcare environments. Innovations in non-real-time AI frameworks offer viable solutions for scaling AI applications in low- and middle-income countries (LMICs), addressing the higher prevalence of HAIs in these regions.

**Conclusions:**

Artificial Intelligence stands as a transformative tool in the fight against hospital-acquired infections, offering advanced solutions for prevention, surveillance, and management. To fully realize its potential, the healthcare sector must prioritize rigorous validation standards, comprehensive data quality reporting, and the incorporation of interpretability tools to build clinician confidence. By adopting scalable AI models and fostering interdisciplinary collaborations, healthcare systems can overcome existing barriers, integrating AI seamlessly into infection control policies and ultimately enhancing patient safety and care quality. Further research is needed to evaluate cost-effectiveness, real-world applications, and strategies (e.g., clinician training and the integration of explainable AI) to improve trust and broaden clinical adoption.

## 1 Introduction

The Centers for Disease Control and Prevention (CDC) define healthcare-associated infections as infections acquired during the provision of healthcare, highlighting the need for effective prevention and control measures (1). Hospital associated infections (HAIs) impose substantial financial burdens on healthcare systems, attributed to extended hospital stays, escalated resource utilization, and additional patient care requirements (2) and represents a substantial and ongoing public health challenge, contributing significantly to morbidity, and mortality (3–5). Despite progress in infection control protocols, HAIs continue to pose a serious threat. The COVID-19 pandemic has further highlighted vulnerabilities in infection control practices, particularly by exacerbating the limitations of traditional surveillance methods, thereby emphasizing the need for innovative solutions. AI, with its ability to analyze vast EHR datasets and integrate unstructured clinical notes, offers a promising avenue for early detection and intervention (6). While primarily a respiratory illness, the pandemic has indirectly influenced the prevalence and management of HAIs. Increased utilization of invasive devices, prolonged hospital stays, and resource constraints have contributed to a rise in HAIs, particularly in intensive care units (ICUs) (7–9).

The estimated HAIs universal prevalence rate is 14%, increasing by approximately 0.06% annually. Regional variations are notable, with the African region experiencing the highest rates at 27%, while the Americas and Western Pacific regions report lower rates around 9% (10). Hospital setting also significantly influences HAIs prevalence, with transplant wards exhibiting the highest rates at 77%, followed closely by neonatal (69%) and ICU (68%) wards (10).

The clinical impact of HAIs is profound, with common infections including surgical site infections (SSIs), ventilator-associated pneumonia (VAP), central line-associated bloodstream infections (CLABSIs), and catheter-associated urinary tract infections (CAUTIs). Infections caused by multidrug-resistant organisms (MDROs) and Clostridioides difficile (CDI) also pose significant risks, contributing substantially to patient morbidity (11).

Each of these infection types carries a high risk of complications and extended recovery times, resulting in increased patient morbidity. SSIs, for example, are among the most frequently reported HAIs and can lead to severe complications such as wound dehiscence and sepsis, particularly in immunocompromised patients (12). Pneumonia, especially VAP, is another significant contributor to HAIs morbidity and mortality and is associated with prolonged ventilator use and higher intensive care unit (ICU) admission rates (13). Bloodstream infections, which are often linked to central line use, can escalate into sepsis, a life-threatening systemic response that greatly increases the risk of mortality (14, 15). Similarly, CAUTIs are frequent among patients with prolonged catheterization (16, 17). Active surveillance strategies are foundational to infection prevention and control efforts aimed at mitigating the burden of HAIs (18). However, these strategies are frequently hindered by the complexity inherent in infection-related datasets and the challenges of monitoring compliance with control measures (19). Traditional surveillance methods, while standard, are often limited by their labor-intensive nature and the need for specialized personnel (19). Traditional surveillance typically involves manual data collection and periodic analysis by healthcare professionals, which can be time-consuming and prone to human error. These limitations underscore the necessity for innovative approaches that enable timely, accurate, and automated infection monitoring. Given the increasing threat posed by HAIs and the rising incidence of antimicrobial resistance (20), there is an urgent need for innovative, scalable approaches. AI offers a transformative solution by enabling real-time, high-precision analyses of vast datasets, identifying infection patterns, and predicting risk factors that are otherwise challenging to detect. Advanced modeling capabilities hold particular promise in addressing antimicrobial resistance through early detection of resistant pathogens and targeted interventions, thus helping healthcare systems manage HAIs more effectively and alleviate the growing burden on public health (21). Within the hospital context, AI applications are vast, ranging from pathogen surveillance and diagnostics to antimicrobial resistance analysis and clinical decision support (21, 22). Effective deployment of AI in infection control depends on both its predictive accuracy and practical usability in clinical settings. Predictive accuracy metrics, such as sensitivity, specificity, area under the curve (AUC), positive predictive values (PRV) and negative predictive values (NPC) (23), are essential for evaluating an AI model's diagnostic performance and reliability in identifying infection risks. However, for AI to be genuinely impactful, usability factors—such as workflow integration, interpretability, and healthcare provider trust—are equally critical (24). These elements ensure that AI tools not only perform well technically but are also effectively adopted and trusted in real-world healthcare environments. Lessons from the COVID-19 pandemic have demonstrated the potential of integrating advanced technologies into infection control practices. The pandemic provided valuable insights into the transmission dynamics of healthcare-associated infections, particularly through the application of machine learning and network analysis to predict hospital-onset COVID-19 infection. As shown in recent study, incorporating dynamic patient contact networks into predictive frameworks significantly improves infection risk stratification and early intervention capabilities (25). These innovations highlight the need for AI-driven approaches to enhance outbreak preparedness and infection prevention beyond respiratory illnesses. The capacity for real-time surveillance supports early intervention strategies and may significantly enhance patient outcomes. This review, distinguish two main applications of AI in HAIs—AI used for early detection of existing infections, which enables prompt treatment and halts further spread. AI used for predicting which patients are likely to develop infections, allowing for targeted preventive interventions.

## 2 Aim and objectives

The overarching aim of this review was to assess the role of artificial intelligence in enhancing hospital-acquired infection prevention and management by evaluating both its predictive accuracy and its usability in clinical settings. To achieve this aim, the following objectives were defined:

To evaluate the predictive accuracy and diagnostic performance of AI models for HAIs,

To assess implementation challenges—including system integration and clinician trust. Although the review summarizes findings narratively, key metrics such as sensitivity, specificity, and AUC are critically discussed to illuminate model performance.

## 3 Methods

This study was conducted as an integrative review (26), designed to synthesize evidence from existing individual studies, systematic and scoping reviews on the role of AI in HAIs. An integrative review was selected to consolidate high-level evidence across numerous studies, facilitating a comprehensive overview of AI applications in HAIs while identifying gaps in the literature. This approach enables a synthesis of findings across various settings, populations, and methodologies, providing valuable insights into AI's predictive accuracy and usability in clinical contexts.

This integrative review was conducted following PRISMA 2020 guidelines (27) to ensure a rigorous and transparent approach. The review process adhered to a structured methodology encompassing eligibility criteria, search strategy, screening and selection, data extraction, synthesis, and bias assessment. The thematic synthesis approach was chosen to structure and interpret the findings (28). Thematic synthesis encompassing a wide range of studies was chosen, as it allows for the identification and organization of recurring patterns and themes across diverse datasets. Given the varied focus and methodologies within the included systematic reviews, scoping reviews, and individual studies on AI applications in HAIs, thematic synthesis provides a systematic way to distill complex information into coherent, interpretable themes that reflect both depth and breadth (28).

## 4 SPIDER framework for inclusion criteria

To structure the scope and inclusion criteria for this integrative review, a SPIDER framework was applied (29), capturing the range of study designs and the diversity of outcomes related to AI's role in HAIs management (Table 1).

**Table 1:** SPIDER framework used to develop the inclusion criteria for the integrative review.

**SPIDER element**	**Description**
S (Sample)	Hospitalized patients susceptible to Hospital Acquired infections and healthcare professionals involved in prevention and management within hospital settings.
PI (Phenomenon of Interest)	Application of AI-based technologies specifically targeting Hospital Acquired infections prevention, detection, prediction, or management.
D (Design)	Systematic reviews, scoping reviews, and original studies, including randomized controlled trials, cohort studies, observational studies, case-control studies, and qualitative research.
E (Evaluation)	Primary evaluation metrics included predictive accuracy (e.g., sensitivity, specificity, area under the curve) and usability factors (e.g., workflow integration, interpretability, clinician trust).
R (Research type)	Quantitative, qualitative, and mixed-methods studies were considered to ensure a comprehensive synthesis of both technical and practical aspects of AI applications.

### 4.1 Eligibility criteria

Inclusion and exclusion criteria were rigorously defined to ensure a precise and targeted synthesis of evidence in alignment with the review's aim and objectives (Table 2). These criteria were carefully crafted to select studies that provide empirical insights into AI's predictive accuracy and clinical usability in infection control settings, directly supporting the review's central objectives and maintaining high methodological standards.

**Table 2:** Inclusion and exclusion criteria for articles included in the integrative review.

**Inclusion criteria**	**Exclusion criteria**
Original research including quantitative and qualitative studies. Systematic reviews, scoping reviews, randomized controlled trials, cohort studies, observational studies, case-control studies, qualitative research, and case studies.	Theses, editorials, opinion pieces.
Studies involving hospitalized patients or healthcare professionals engaged in HAIs prevention and management within hospital settings.	Studies focused primarily on community-acquired infections or conducted only in non-hospital settings (e.g., outpatient clinics, community care environments).
Studies using AI technologies specifically for HAIs prevention, detection, prediction, management, or surveillance, with a focus on predictive accuracy and practical usability within clinical workflows.	AI applications not directly related to infection control, predictive accuracy, or usability in infection prevention or management settings.
Studies reporting on predictive accuracy metrics [e.g., sensitivity, specificity, area under the curve and usability outcomes (e.g., workflow integration, interpretability, provider trust, clinical decision support)].	Studies lacking relevant outcomes, such as predictive accuracy, diagnostic performance, or usability factors crucial to the practical application of AI in infection control.
Studies published between July 2014 and July 2024, with an updated search in November 2024 to capture recent publications.	Studies published before July 2014.

### 4.2 Search strategy

A detailed search strategy was employed across multiple databases (MEDLINE/PubMed, Embase, IEEE Xplore, CINAHL, and SCOPUS), using specific keywords and filters such as “artificial intelligence,” “machine learning,” “deep learning,” “hospital-acquired infections,” “nosocomial infections,” “infection prevention,” and “surveillance”—for literature published from July 2014 to July 2024, with a follow-up search in February 2025 to identify any new relevant studies. The search strategy, developed with a research librarian, targeted both AI's predictive accuracy and usability within clinical workflows for HAIs prevention and management. Search terms were structured to encompass artificial intelligence, machine learning concepts, HAIs, predictive accuracy, and usability factors (Table 3). Boolean operators and synonyms were utilized to ensure thorough retrieval of relevant studies across databases (27).

**Table 3:** Search terms utilized during the literature search phase of the integrative review.

**Concept**	**Keywords**
Artificial intelligence (AI) and machine learning concepts	“artificial intelligence” OR “AI” OR “machine learning” OR “deep learning” OR “predictive modelling” OR “algorithm” OR “data mining” OR “natural language processing”
Hospital-acquired infections (HAIs)	“hospital-acquired infection” OR “nosocomial infection” OR “healthcare-associated infection” OR “HAI” OR “infection prevention” OR “infection control” OR “pathogen detection” OR “surveillance”
Predictive accuracy and diagnostic performance	“predictive accuracy” OR “diagnostic performance” OR “sensitivity” OR “specificity” OR “area under curve” OR “AUC” OR “precision” OR “recall” OR “ROC curve” OR “classification” OR “prediction”
Usability and practical application	“usability” OR “practical application” OR “implementation” OR “integration” OR “clinical usability” OR “workflow integration” OR “clinical decision support” OR “trust in AI” OR “interpretability” OR “adoption” OR “acceptance”

### 4.3 Selection process

The records identified through database searches were imported into Rayyan, a systematic review screening tool (30), where duplicates were removed. Two independent reviewers (RA and FA) screened all resulting titles and abstracts based on eligibility criteria. Full-text articles were subsequently evaluated for final inclusion. Any discrepancies in study selection were resolved through discussion, with a third reviewer (JS) involved to reach consensus where necessary.

### 4.4 Data extraction and synthesis

Data extraction was conducted independently by two reviewers, focusing on study characteristics, AI model type, predictive accuracy metrics (e.g., sensitivity, specificity, AUC), usability factors (e.g., workflow integration, interpretability, and clinician trust). Discrepancies were resolved through consensus. Data were synthesized thematically, capturing recurring themes relevant to objectives (31).

### 4.5 Quality assessment of included studies

The included studies were assessed for methodological quality using standardized tools appropriate to their design. Predictive modeling studies were evaluated using the TRIPOD checklist (32) to assess reporting transparency and methodological rigor. Quality was primarily assessed using the AMSTAR 2 tool for review articles, while additional criteria were applied to non-systematic reviews of predictive modeling studies (33).

## 5 Results

**Figure 1:**
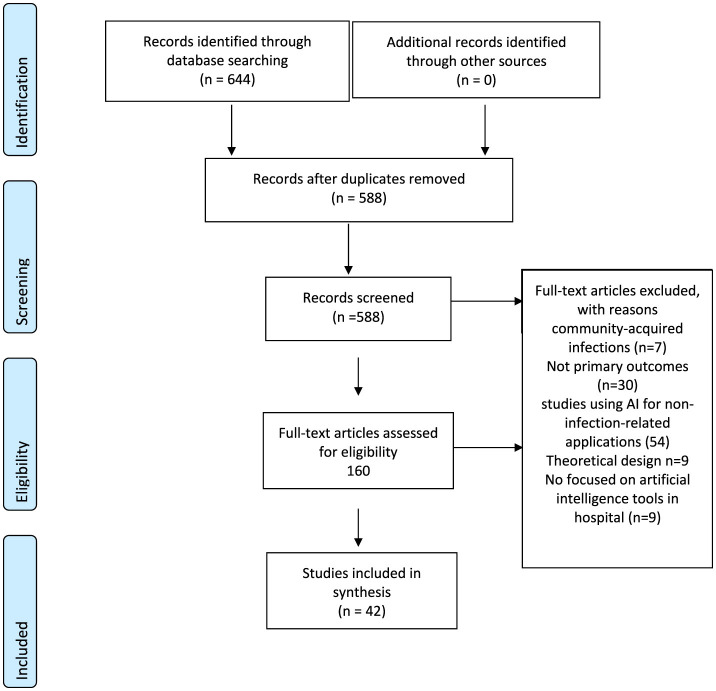
PRISMA flow diagram.

The search strategy yielded a total of 588 studies across all databases, which was subsequently refined to a total of 42 studies eligible for inclusion in the final dataset (Figure 1).

### 5.1 Characteristics of study

This review synthesizes 37 individual studies (Appendix A1) from Brazil (34, 35), Cambodia (36), Norway (37), Canada (38–40), China (41–45), Denmark (46–49), Italy (50–52), Japan (53), Pakistan (54), South Korea (55, 56), Spain (57), Taiwan (58), and the United States (59–70). Additionally, three systematic reviews (22, 71, 72) and two scoping reviews (73, 74) were included (Appendix A2). Many individual studies that relied on data from specific hospitals used data from single institutions or departments (34, 36, 38, 39, 41–45, 50, 53–56, 58, 61, 62, 64, 65). Additionally, the primary studies were systematically classified into Model Development or Implementation and Evaluation studies (Appendix A3).

### 5.2 Quality assessment

This integrative review included 42 studies: two scoping reviews, three systematic reviews, and 37 individual studies. Each included study was meticulously evaluated using TRIPOD (Transparent Reporting of a Prediction Model for Individual Diagnosis) for predictive studies and AMSTAR 2 (A Measurement Tool to Assess Systematic Reviews) for the reviews. The TRIPOD framework is essential to ensure consistency, transparency, and reproducibility across predictive modeling studies, particularly in high-stakes healthcare applications like infection prevention and control. The quality assessment here reflects the adherence of all 37 individual studies to TRIPOD standards across key domains: study objectives, data sourcing, participant criteria, predictor transparency, outcome definition, model development and validation, and performance interpretation (32) (Appendix A4).

All included studies clearly articulated their objectives, with each aiming to enhance infection prediction, or intervention strategies through predictive modeling. Studies exemplified this clarity, contributing clear goals relevant to infection risk assessment or management in clinical setting. All the 37 studies presented adequate descriptions of their data sources, often drawing from robust electronic health records (EHRs) or large surveillance databases, essential for ensuring data quality and representativeness. Participant selection varied across studies, with some studies providing extensive inclusion and exclusion criteria, while others offered more limited demographic or clinical specifics. Studies clearly detailed inclusion and exclusion criteria, contributing to a thorough understanding of the populations targeted (39, 41, 44, 50, 66). In contrast, other studies would benefit from enhanced reporting on participant demographics, comorbidities, or other selection details (49, 59). This level of transparency is essential for assessing generalizability and ensuring that predictive models are applicable across diverse patient groups.

Most studies demonstrated solid adherence to TRIPOD guidelines by detailing the predictors used, often encompassing a mix of clinical, demographic, and procedural variables. In several cases (55, 70), specific feature selection techniques, such as recursive feature elimination (RFE) and feature importance rankings (i.e., metrics that quantify the contribution of each predictor to the model's predictions), were used to enhance model interpretability. However, studies like Hopkins et al. and Lind et al. (60, 62) could further improve by providing explicit lists of all predictors used, which would strengthen transparency and facilitate application in diverse clinical settings. Consistent outcome definitions, aligned with clinical guidelines, were reported in all studies, ensuring clear identification and reproducibility of endpoints.

Model development and validation processes were generally robust across the included studies, with most employing cross-validation or external validation techniques to test model reliability. For example, studies by Jakobsen et al., Zachariah et al., Hopkins et al., Mamlook et al., and Scardoni et al. (47, 59, 60, 67, 70) provided in-depth details on their model validation processes, including data split ratios, cross-validation methods, and hyperparameter tuning. Studies like those by Cho et al. and Caglayan et al. (53, 64) could further improve by including additional specifics regarding validation techniques, particularly in complex clinical models. Performance metrics such as AUC, sensitivity, specificity, and accuracy were consistently reported across the studies, enabling an effective assessment of model predictive power. Additionally, several studies incorporated feature importance rankings or interpretation aids, such as SHAP values, as seen by Møller et al., and Scardoni et al. (48, 70), which enhance clinical interpretability by identifying the most influential predictors.

The AMSTAR 2 tool, a widely recognized instrument for assessing systematic was applied to evaluate the methodological rigor of the included reviews (33) (Appendix A5–A9). clarity, We applied the full AMSTAR 2 tool to the three systematic reviews, while for the two scoping reviews we used an adapted version of the AMSTAR 2 tool, tailored to the objectives of scoping reviews. This assessment revealed both strengths and limitations, particularly in terms of study selection, bias management, and transparency, which are crucial for advancing AI applications in HAIs. In evaluating the methodological rigor of included scoping reviews (73, 74), an adapted version of the AMSTAR 2 tool was employed to ensure a comprehensive and high-quality assessment. Recognizing the unique purpose of scoping reviews, which aim to map evidence and identify gaps rather than synthesize outcomes (75), we selectively applied AMSTAR 2 criteria that align with these objectives. This approach focused on aspects of methodological transparency and comprehensiveness. The overall quality rating of the five reviews included in this analysis reflects a solid, though varied, adherence to methodological rigor. Four of the reviews (22, 72–74) demonstrated moderate quality, meeting essential criteria for transparent objectives, and clear data extraction processes. The systematic review by Scardoni et al. (71) was rated as moderate to high quality due to its rigorous adherence to PRISMA guidelines, comprehensive search strategy, and independent data extraction. Its methodological strength, particularly in addressing heterogeneity and transparently reporting finding.

### 5.3 Results

Our thematic synthesis revealed two major domains: predictive performance and clinical usability. Although many models demonstrated AUCs above 0.80, these results often derive from single-center, retrospective studies with inherent limitations. Comparative analyses reveal that deep learning models sometimes outperform traditional machine learning algorithms in handling high-dimensional and complex datasets, though both approaches show strong potential.

### 5.4 Predictive accuracy of AI models for HAI detection and classification

One of the most critical indicators of AI model effectiveness in HAIs management is predictive accuracy. Predictive accuracy refers to the ability of an AI model to correctly identify or forecast the presence or risk of an infection, as measured by metrics such as sensitivity, specificity, and the area under the receiver operating characteristic curve (AUC). Various studies employing machine learning (ML) and natural language processing (NLP) techniques. Across these studies, models targeting specific infections, such as SSIs, urinary tract infections (UTIs), consistently demonstrated high accuracy metrics, notably AUC scores and predictive sensitivity.

1. **Surgical Site Infections (SSIs):** This subtheme presents how AI is used to predict and detect SSIs, which are common and critical post-surgical complications. Different AI models are applied to improve prediction accuracy for SSIs.
  A subset of Sohn et al., Hopkins et al., Petrosyan et al. (38, 59, 61) focused on detecting SSIs following surgical procedures. For example, Zachariah et al. (59) employed a Bayesian network model enriched by NLP for SSIs detection post-colorectal surgery, achieving an AUC of 0.892 and indicating enhanced accuracy when surgeon-defined criteria were applied. Hopkins et al. (61) applied a deep neural network model to predict SSIs in patients undergoing spinal fusion surgeries, yielding an AUC of 0.775 and high PPV and NPV, suggesting effective clinical applicability for SSIs prevention. Additionally, Petrosyan et al. (38) achieved an AUC of 0.91 for SSIs prediction within 30 days post-surgery, demonstrating the model's strong calibration and suggesting its potential utility in high-risk surgical settings. The review by Radaelli et al. (72) confirm that models for SSIs, like Random Forest and Bayesian networks, achieve high area under the receiver operating characteristic (AUROC) scores, reinforcing AI's precision in detecting infections in postoperative care.
2. **Urinary Tract Infections (UTIs):** This subtheme addresses the application of AI in predicting UTIs, with a focus on hospital admission data and patient histories.
  Studies like Zachariah et al., Møller et al., and Jakobsen et al. (48, 49, 60) illustrated AI's capacity to predict UTIs with robust performance across different methodologies. Zachariah et al. (60) applied decision tree and neural network models to identify UTI risk at hospital admission, reporting a sensitivity of 78.2% and specificity of 64.2% for the decision tree model and contrasting specificity and sensitivity profiles for the neural network model. Møller et al. (49) and Jakobsen et al. (48) demonstrated the utility of clinical data and historical health information in decision tree and neural network models, achieving AUC of 0.81 and 0.758, respectively, further reinforcing AI's utility in UTIs prevention.
3. **Broader HAIs Detection and Surveillance:** This subtheme highlights the use of AI models designed to predict and monitor a wide range of HAIs, such as VAP, and CLABSI. These models are like multi-purpose tools that can address multiple types of infections within a single system, making them valuable for general infection surveillance in high-risk settings like ICUs.
  Several studies assessed AI models for general HAIs detection. Dos Santos et al. (34) developed a neural network model trained on EHRs data over 18 months, reporting a ROC AUC of 0.903, with 88.57% sensitivity and 90.27% specificity, underscoring the model's ability to effectively classify HAIs in high-risk patients. Barchitta et al. (51) focused on ICU and integrated AI with the Simplified Acute Physiology Score (SAPS II), achieving an AUC of 0.90, suggesting AI's capability in amplifying traditional scoring methods to enhance infection risk stratification. The review by Baddal et al. (22) highlights the application of AI models, including random forests, logistic regression, and deep learning techniques, for predicting infections such as VAP and CLABSIs. In some studies, high-performing models achieved AUC scores between 0.76 and 0.85 for early VAP prediction, demonstrating the potential of ML in infection risk stratification and management. Similarly, Scardoni et al. (71) observed that AI-based models, especially those using ML, often outperformed traditional statistical methods, achieving high specificity, sensitivity, and AUC scores across various HAIs. For instance, a random forest model for CLABSIs prediction achieved an AUC of 0.87, underscoring AI's effectiveness in infection risk stratification. Zhang et al. (73) focused on AI models for VAP, noting an average AUC of 0.86 across different ML algorithms, with random forest models showing particularly strong predictive performance. This reinforces the effectiveness of AI in early HAIs detection, especially for high-risk ICU patients.
4. **Other Infection-Specific Models:** This subtheme focuses on AI applications specifically developed for managing single infections, such as VAP, or MDRO colonization. These models are like specialized tools, designed to address the unique clinical needs of individual infections. By providing highly tailored predictions, they support more precise decision-making in critical situations
  The review included studies focusing on sepsis (63) ventilator-associated infections (69), and MDRO colonization (65). For instance, Lind et al. (63) employed two automated ML systems trained on EHR data for predicting sepsis risk, achieving sensitivities of 80% and 65.7% and specificities of 72.8% and 66.9%. In the context of bloodstream infections (BSIs), the study by Bopche et al. (37) highlights the feasibility of non-real-time AI models, which leverage historical EHR data. This approach not only demonstrates high predictive accuracy but also offers scalability for broader implementation in hospitals with limited access to real-time monitoring systems. Caglayan et al. (65) developed a model for predicting MDRO colonization upon ICU admission, attaining an AUC of 0.83, reflecting AI's precision in early identification of MDRO cases to prevent the spread of resistant pathogens. The review by Bomrah et al. (74) demonstrates that machine learning models, particularly Random Forest and XGBoost, consistently achieved high AUROC values for sepsis prediction, outperforming traditional scoring systems. These findings highlight the potential of machine learning for timely and accurate sepsis detection in ICU and emergency settings. Additionally, literature reviews by Baddal et al. (22) and Scardoni et al. (71) report that AI models show promise in reducing hospital stays and mortality rates, underscoring their predictive and preventive value in clinical applications. Zhang et al. (73) further emphasizes the frequent use of machine learning algorithms, including random forests and neural networks, in AI models for VAP, pointing to high accuracy and AI's critical role in stratifying patient risk.
5. **Predictive Value Across Diverse AI Algorithms:** This subtheme, focuses on the comparative predictive value of different ML algorithms for detecting infections like VAP, emphasizing the overall performance of algorithms (especially ensemble models and deep learning).
  Different ML algorithms were evaluated across studies, including logistic regression, neural networks, decision trees, Bayesian networks, and ensemble models. Studies indicated that deep learning models and ensemble approaches often achieved superior predictive accuracy, particularly in complex datasets with high-dimensional data. Notably, Flores-Balado et al. (57) reported an AUC of 0.989 for detecting SSIs post-hip replacement using an NLP-based gradient boosting model, and Zhu et al. (44) found that ensemble learning models outperformed single algorithmic approaches for UTIs prediction in immobile stroke patients. According to the review by Bomrah et al. (74), robust feature engineering, including methods like filter, wrapper, and embedded approaches, significantly enhances ML model accuracy. The review highlights that Random Forest and XGBoost models, optimized with critical features such as vital signs and lab values, achieved sensitivity improvements essential for early infection risk assessment.

### 5.5 Usability and practical integration of AI tools in clinical settings

In addition to predictive accuracy, practical considerations surrounding the usability and integration of AI tools in clinical environments emerged as crucial for AI adoption. Several studies addressed factors such as interpretability, clinician trust, and workflow compatibility, which influence the operational value of AI in infection control.

1. **Interpretability and transparency:** interpretability is essential for gaining clinician trust and ensuring AI predictions are actionable. various studies leveraged explainable AI techniques, such as SHapley additive explanations (SHAP) values, to elucidate model predictions. for instance, lee et al. (56) utilized SHAP values to demonstrate feature importance in predicting antibiotic resistance patterns, allowing clinicians to understand which variables—such as prior antibiotic use and demographic factors—influence infection risk. Jakobsen et al. (47) similarly adopted Bayesian networks incorporating clinical expert knowledge, which provided interpretable and clinically intuitive predictions, enhancing the model's suitability for infection risk stratification in HA-UTI management.
2. **Integration with Existing EHR Systems:** Studies consistently highlighted the importance of seamless integration with EHRs to ensure timely access to data and facilitate real-time prediction. Bonde et al. (46) demonstrated successful NLP-based model integrations with EHRs chart notes, improving the detection of superficial SSIs and postoperative infections. These integrations were shown to reduce the need for manual reviews significantly (40, 46, 55, 57), as Cho et al. (55) reported a potential reduction in chart reviews by 83.9% using an AI model combined with a rule-based algorithm, highlighting the operational efficiency gains possible with AI. The review by Baddal et al. (22) underscores the critical role of data integration from EHRs in ensuring the accuracy and functionality of AI-based infection surveillance systems. Access to real-time EHR data significantly enhances the predictive power of AI models, facilitating timely infection detection and response within clinical workflows. Reviews by Radaelli et al., and Scardoni et al. (71, 72) similarly highlight EHRs as the primary data source, reinforcing the importance of comprehensive data integration for optimizing AI performance in HAIs detection. In contrast, Zhang et al. (73) points out that most VAP prediction models draw data from public databases like MIMIC-III, which primarily include structured clinical and laboratory data. This review also notes the absence of imaging data, suggesting that addressing this gap could further improve model accuracy.
3. **Scalability and Adaptability Across Settings:** AI tools' generalizability across various clinical settings and infection types is vital for widespread adoption. While models trained in single-center studies showed strong internal validation, multicenter studies such as Zhu et al. (44) indicated that AI models could be generalizable across different hospitals, enhancing their potential for broader clinical utility. Additionally, studies like Huang et al. (45) underscored AI's adaptability by implementing an AI-based training and monitoring system (AITMS) across multiple departments, which improved compliance with personal protective equipment (PPE) protocols and decreased infection rates, demonstrating the potential for cross-functional applications of AI in infection prevention.
4. **Challenges in Model Deployment and Clinical Implementation:** Despite promising results, several studies identified practical barriers to the implementation of AI tools. Challenges included model interpretability, data quality, and the need for clinician training on AI-driven decision support systems. For example, Rennert-May et al. (39) and Walker et al. (64) reported difficulties in translating model predictions into clinical actions due to the complexity of algorithmic outputs and the need for user-friendly interfaces that align with clinical workflows. As highlighted in recent reviews (22, 71, 72), challenges—including model variability, data quality, risk of bias, the need for standardized protocols, high costs, healthcare worker resistance, and limited evidence on real-world impact—continue to hinder broader implementation efforts. While reviews of VAP prediction models, such as Zhang et al. (73), note promising performance, they also underscore that studies have largely focused on internal validation without real-world application. Further challenges, including the lack of external validation, integration with clinical workflows, and exclusion of nurse-related data, remain barriers to adoption. Likewise, Bomrah et al. (74) emphasize in their review of sepsis prediction models the need for standardized feature engineering and external validation to ensure reliability and clinical integration.

## 6 Discussion

The findings of our integrative review suggest that AI has substantial potential to enhance HAI prevention through early detection and optimized infection control strategies. While many models report high predictive accuracy, these results are often tempered by methodological shortcomings such as reliance on retrospective data and internal validation. Traditional approaches to infection control often rely on manual surveillance and retrospective analyses, whereas AI introduces a paradigm shift toward proactive, real-time risk assessment and decision-making (24, 76). Our findings contribute to this growing body of literature by demonstrating that AI's predictive capabilities, when integrated into healthcare workflows, could address longstanding challenges in HAIs management.

### 6.1 Enhanced surveillance and early detection in HAIs control

Surveillance strategies play a critical role in preventing, diagnosing, and managing HAIs, with substantial literature advocating for the integration of emerging technologies to advance these efforts (35). AI developments now offer a transformative potential for HAI surveillance, enabling more precise decision-making (19). Broad evaluations in the field affirm AI's value in enhancing HAIs control, supporting findings from this integrative review. A systematic review highlighted AI-driven approaches that show promise in HAI monitoring and surveillance (71). These advancements indicate a potential paradigm shift in HAIs surveillance, carrying significant implications for future research and strategic integration within infection control frameworks.

Our review emphasizes the integration of unstructured data, such as clinical notes, with structured EHR data to improve HAIs surveillance. Studies, such as that by Shi et al. (77), demonstrate that using NLP to extract infection-related information from clinical notes combined with structured data enhances machine learning models' specificity and predictive accuracy for surgical site infections. Their approach, utilizing a random forest model with high specificity and positive predictive value, suggests that NLP-augmented AI models may overcome limitations in traditional manual chart reviews, which are resource-intensive and prone to underreporting. Nonetheless, challenges remain in interpreting negations and contextual nuances within clinical notes, indicating a need for more sophisticated temporal and context-aware features to optimize AI's role in infection control.

AI's role in infection surveillance, particularly through ML and NLP, is critical for early HAIs detection. These technologies enable healthcare systems to identify infections before they become clinically evident. Our review findings demonstrate that AI-driven models are particularly effective in continuously analyzing patient data streams, providing real-time risk assessments and reducing reliance on labor-intensive manual reviews. This aligns with broader findings showing that AI can process both structured and unstructured data—including EHRs entries, clinical notes, and diagnostic images—to achieve high sensitivity and specificity in infection detection (78).

### 6.2 Integration challenges and the necessity of clinician trust

AI-driven surveillance systems promise to alleviate the significant clinical burden associated with traditional HAIs monitoring, thereby allowing clinicians to focus on critical infection control interventions. This shift has profound implications for healthcare resource allocation, as automated systems reduce the need for manual chart reviews, freeing clinical staff for more direct patient care. As noted by Wolfensberger et al. (79), fully automated systems can achieve time and cost savings while maintaining or even enhancing infection detection accuracy. This review reaffirms that AI-driven infection monitoring can streamline clinical workflows, which is crucial for high-demand environments such as ICU, where HAIs are prevalent.

Despite AI's promising contributions to infection control, several significant barriers to implementation remain, including model interpretability, data standardization, and the need for clinician trust. The lack of standardized data formats limits AI model generalizability across institutions and hinders EHRs integration. To maximize AI's utility, healthcare systems need interoperability solutions that facilitate seamless data exchange, thereby enhancing model accuracy and reliability. The findings of this review highlight that consistent data standards and EHRs compatibility are foundational to AI's broader clinical applicability.

Clinician trust is paramount for AI adoption, as clinicians must rely on AI insights to make critical care decisions. Explainable AI (XAI) methods, such as SHapley Additive exPlanations (SHAP), show promise in making model predictions more transparent by identifying key predictive variables, thus improving clinician understanding and confidence in AI-generated insights (56). While XAI methods aim to enhance interpretability, it is crucial to go beyond these frameworks to ensure AI-driven insights align with traditional diagnostic practices and clinical expertise. However, current literature lacks robust evidence on clinicians' trust in existing AI models, underscoring the need for qualitative research to explore their experiences, perceptions, and concerns. Such research can provide critical insights into barriers and facilitators of trust, offering a foundation for designing AI tools that align with clinical workflows. Additionally, user-friendly interfaces and tailored training programs remain important components in fostering trust and encouraging adoption, but their design should be informed by the findings of qualitative studies.

## 7 Methodological discussion

A pivotal methodological observation in this integrative review is the inconsistency in validation techniques across studies evaluating AI models for HAIs prediction. Validation practices play a central role in ensuring that performance metrics—such as AUC, sensitivity, and specificity—accurately represent a model's generalizability beyond its training data. Variability in these practices, however, risks distorting true model performance, hindering reliable comparisons and potentially overstating or understating the models' predictive power.

For instance, studies relying on internal validation methods, such as cross-validation, may present more favorable metrics due to potential overlaps between training and test data, potentially leading to an optimistic assessment of model accuracy. Conversely, studies employing external validation, which uses datasets from different populations or healthcare environments, often yield slightly lower metrics, yet provide a more realistic perspective on a model's robustness and generalizability in diverse clinical settings.

This inconsistency underscores the need for a more standardized approach to validation and reporting in future studies. Establishing methodological transparency and consistent performance metrics is essential to accurately evaluate and compare AI models. Such rigor will enable the field to identify models that reliably translate to real-world clinical settings, ultimately supporting AI's role in advancing infection control and prevention.

## 8 Strengths and limitations

This integrative review provides a comprehensive synthesis of AI applications in HAIs, drawing from a wide range of high-quality sources, including systematic reviews, scoping reviews, and individual studies. The use of validated tools, such as the TRIPOD and AMSTAR 2, enhances the methodological rigor of this review by ensuring consistent reporting quality and minimizing biases. This study is strengthened by the thematic synthesis approach, which captures the depth and breadth of AI's predictive capabilities and usability within clinical contexts. By including diverse AI model types and infection types, this review provides a holistic perspective that highlights AI's potential for reducing HAIs and addresses key factors influencing its clinical applicability.

However, some limitations must be acknowledged. First, while the review spans multiple AI applications, the heterogeneity of the included studies—covering various infection types, AI algorithms, and clinical settings—introduces variability that may limit the generalizability of certain findings. Furthermore, the majority of studies rely on retrospective data, which could lead to selection biases and impact the predictive accuracy of AI models in real-time clinical settings. The reliance on EHRs data, which varies in quality and completeness across institutions, also poses a limitation. Additionally, this review's focus on predictive accuracy and usability may overlook other important factors, such as cost-effectiveness and long-term impacts on patient outcomes, which would be valuable to explore in future research.

**Table:** How AI tools work (lay summary).

AI models analyze large datasets (e.g., electronic health records) to identify patterns associated with infections.	They use complex algorithms to detect subtle signals in patient data that may indicate the onset of an infection.	This process enables earlier detection, timely intervention, and improved patient outcomes by guiding clinical decisions.

## 9 Recommendations and implications

To support AI integration in HAIs management, healthcare administrators must prioritize investments in data infrastructure, interoperability, and clinician training. Policies should encourage the adoption of EHRs that can seamlessly integrate AI-driven tools, allowing real-time data utilization and enhancing infection control capabilities.

Establish clear guidelines ensuring patient data privacy and ethical AI use. Policymakers can develop frameworks that regulate data sharing across healthcare systems while maintaining patient confidentiality, fostering a trusted AI implementation environment.

Clinicians should advocate for AI models that provide interpretable outputs to enhance their practical application in infection control. Models using SHAP or similar interpretative methods allow clinicians to understand risk factors more transparently and support decision-making processes.

Infection control teams can benefit by incorporating AI predictions into daily surveillance protocols. AI models can serve as an adjunct to traditional infection surveillance, enabling earlier detection and intervention, particularly for high-risk units such as ICUs and surgical wards.

IT departments should focus on ensuring data quality and consistency in healthcare records, which are critical for accurate AI predictions. Standardizing data collection methods and implementing regular data audits can enhance the reliability of AI-driven models.

Developing scalable and flexible AI systems that work across different hospital settings will support multicenter collaborations. Such initiatives can also aid in the external validation of AI tools, increasing their generalizability and reliability across diverse clinical environments.

AI developers must emphasize external validation to ensure the adaptability of AI models in different clinical contexts. Including usability testing with end-users, such as infection control professionals, will help tailor AI tools to fit specific workflows and improve adoption rates.

AI researchers should develop ethical frameworks that address potential biases in AI models, especially when using diverse demographic data. Techniques such as fairness-aware machine learning can mitigate bias, ensuring equitable AI-driven healthcare solutions.

Future research should focus on large-scale, longitudinal studies across multiple hospital systems to validate AI models and improve their reliability in varied clinical settings. Multicenter studies will also enhance the model's ability to handle heterogeneous data, which is common in healthcare.

Advanced AI techniques, including ensemble learning and deep learning, should be further explored to tackle complex HAIs, such as multidrug-resistant infections. Developing models tailored to specific HAIs and patient populations can lead to more precise interventions.

Comprehensive assessments of AI's cost-effectiveness in reducing HAIs and its impact on clinical outcomes are essential. These studies can provide data to justify the financial investment in AI-driven infection control, making the case for broader adoption across healthcare systems.

To improve usability, AI research should prioritize the development of interfaces designed around the workflows of infection control teams and clinicians. User-centered design in AI tools can enhance clinical acceptance and reduce the learning curve associated with adopting new technologies.

## 10 Conclusion

This integrative review highlights the potential of AI to transform HAIs prevention by enhancing early detection and supporting infection control efforts. AI models, particularly those integrated with electronic health records, have demonstrated high accuracy in identifying infections such as surgical site infections and urinary tract infections. These predictive tools can guide timely interventions, reducing the burden of HAIs on healthcare systems. However, broader adoption requires further validation of these models across diverse healthcare settings, simplified integration into clinical workflows, and clinician-friendly interpretability features. By addressing these challenges, clinicians and administrators can leverage AI to strengthen patient safety and infection control.

## Data Availability

The original contributions presented in the study are included in the article/Supplementary material, further inquiries can be directed to the corresponding author.
