# Supplementary material for: Artificial intelligence in hospital infection prevention: an integrative review
Source: Front Public Health. 2025 Apr 2;13:1547450. doi: 10.3389/fpubh.2025.1547450 (PMC12001280; doi:10.3389/fpubh.2025.1547450)
Supplement: Supplementary file 1 [file Data_Sheet_1.docx]

**Appendix Table A1: *Characteristics of Included Individual Studies in the Umbrella Review***

| **Author and date** | **Setting** | **country** | **Study design** | **Sample** | **AI tool and methodology** | **Type of infection** | **Findings** |
| --- | --- | --- | --- | --- | --- | --- | --- |
| Rajeev Bopche et al., 2024 | St. Olavs Hospital, Norway | Norway | Retrospective cohort | 35,591 patients | Explainable AI framework using tree-based models (e.g., Random Forest, XGBoost, CatBoost). Historical EHRs analyzed. | Bloodstream Infections | Explainable AI framework demonstrated superior specificity and sensitivity compared to traditional models. It leverages historical EHRs for early prediction without requiring real-time data, promising better resource allocation and outcomes |
| (Simioli et al., 2024) | Respiratory Pathophysiology and Rehabilitation Unit, Monaldi Hospital, Naples | Italy | Observational study | 1704 hand washing events evaluated | AI-driven "Soapy Clean Machine" - an intelligent hand-washing station monitoring technique, step-by-step guidance, adherence scoring, feedback mechanism, and a cloud platform for real-time reporting | Multiple (HAIs) | Findings show 74% successful compliance with hand hygiene steps. The machine highlights recurring issues in the sequence and suggests further attention to sequence accuracy and training needs. |
| (Lukasewicz Ferreira et al., 2024a) | Tacchini Hospital | Brazil | Comparative observational study | 6,296 patients from July 2021 to December 2021. | The study used a random forest algorithm in a semiautomated AI-based surveillance system | Multiple (HAIs) | Manual Surveillance identified 183 HAIs cases 2.9% while the ML-based semiautomated method identified 4.7%. The semiautomated ML enhance HAIs surveillance accuracy and efficiency, supplementing traditional methods. |
| (Sohn et al., 2017) | Three acute care facilities in New York City | United States. | Retrospective study | 751 colorectal surgery cases | Bayesian network identifying risk factors from a quality improvement programme and key words from clinical notes | SSIs | SSI detection showed a ROC AUC of 0.827, increasing to 0.892 with surgeon-defined clinically meaningful SSIs |
| (Zachariah et al., 2020) | Hospital | United States | retrospective cohort study | 897,344 adult hospitalizations. | ML models (neural networks and decision trees) utilising admission data from EHRs | UTIs | Decision tree model showed higher sensitivity vs neural network model (78.2% vs 57.3%) and lower specificity (64.2% vs 81.4%); PPVs and NPVs were 3.5% and 99.4% for the decision tree and 4.9% and 99.1% for the neural network, respectively |
| Hopkins et al. (2020) | Single academic center, specifically the Departments of Neurological Surgery and Orthopedic Surgery. | United States | Retrospective cohort study | Patients undergoing posterior spinal fusion surgery | ML model trained on data from 4,046 patients | SSIs | The ML predicted SSIs with an AUC of 0.775, PPV 92.56% and NPV 98.45%; the model also identified risk factors and protective factors |
| (Kanjilal et al., 2020) | Single academic center using electronic health record (EHR) data | United States | Retrospective cohort study | 3,629 patients presenting with uncomplicated UTIs between 2014 and 2016 | ML model on antibiotic recommendations | UTI antibiotics | recommended first-line, narrow-spectrum antibiotics more effectively than clinicians, resulting in a 67% reduction in second-line antibiotic use compared to clinician-prescribed treatments. |
| (Barchitta et al., 2021) | ICU department | Italy, from the 'Italian Nosocomial Infections Surveillance in Intensive Care Units' project. | Retrospective cohort study | 7,827 ICU patients. | Machine learning model for the risk of ICU-associated infections | HAI | The model achieved an AUC of 0.90 when combined with existing risk stratification tools |
| (dos Santos et al., 2021) | Hospital Tacchini, a general hospital | Brazil | Retrospective cohort study | All admitted patients | AI model intended to monitor HAIs derived from 18 months of data (5,105 patients) | automated HAI surveillance. | The model (neural network) had a ROC AUC of 0.903, sensitivity 88.57% and specificity 90.27%; the model correctly classified 67/73 patients with HAIs |
| (Lind et al., 2021) | Fred Hutchinson Cancer Research Center, Seattle, Washington | United States | Retrospective cohort study | Recipients of allogeneic haematopoietic cell transplant | Two automated ML systems using EHR data to predict sepsis, learning from EHR data on 1,943 patients who previously received transplants | Sepsis / bacteraemia | Both systems were sensitive (80% and 65.7%) and specific (72.8% and 66.9%) in predicting risk of sepsis and bacteraemia |
| (Møller et al., 2021) | Hospital setting, using EHRs to gather patient data. | Southern Denmark | Retrospective cohort study | All admitted patients | Decision tree models to predict UTI based on admission data and historical data, trained on demographics, laboratory results, antibiotic treatment data and clinical data from 301,932 patients | HAIs UTI | Both models had high ROC (0.74 and 0.81) with high sensitivity and specificity; models could personalise UTI prevention strategies based on admission data |
| (Petrosyan et al., 2021) | Ottawa Hospital, Canada. | Canada. | Retrospective cohort study. | 14,351 patients who underwent surgery, of whom 795 (5.5%) developed an SSI. | ML model for the prediction of SSIs within 30 days of surgery | SSIs | The model showed an AUC of 0.91 and strong calibration. |
| (Walker et al., 2021) | A single tertiary care pediatric hospital | United States | Retrospective cohort study | 969 CLABSI events from 453 distinct pediatric patients over a 14-year period | the study applied four machine learning models, aiming to predict two outcomes: infection recurrence and CVC removal. | (CLABSIs) in pediatric patients with CVCs. | Model performance varied by time and outcome, with the best-performing models achieving AUROC scores between 0.56 and 0.83. |
| (Wang et al., 2021) | Taizhou Central Hospital | China | Retrospective cohort study | 705 adult patients who underwent minimally invasive surgery from May 2012 to October 2019 | Six machine learning algorithms were applied, clinical data and laboratory tests | (SSIs) within 30 days post-surgery. | The Naïve Bayes model achieved the highest performance, with an AUC of 0.78, sensitivity of 0.93, specificity of 0.82, and accuracy of 0.90. |
| (Caǧlayan et al., 2022) | Intensive Care Units (ICUs) at the University of Maryland Medical Center. | United States | Retrospective cohort study | 4,670 ICU admissions from 3,958 patients over two years | Three machine learning algorithms to predict colonization with multidrug-resistant organisms (MDROs) upon ICU admission | Colonization with MDROs | The Random Forest model achieved 82% sensitivity and 83% specificity for MDRO colonization prediction, supporting use of models as a clinical decision support tool in high-risk patients |
| (Li et al., 2022) | A single medical center in northeast China. | China | Retrospective cohort study | 246 hospitalized patients with invasive candidal and bacterial bloodstream infections, admitted between January 2013 and January 2018 | Three machine learning models, the dataset was split into training and test sets in a 7:3 ratio | Invasive candidal infections with bacterial bloodstream infections. | The Random Forest model achieved the best performance, with an AUC of 0.919, indicating high accuracy in predicting in-hospital mortality risk |
| (Liu et al., 2022) | First Affiliated Hospital of Nanchang University | China | Retrospective cohort study | 288 patients who underwent LSS between December 2010 and December 2019, with 144 developing SSI and 144 not developing SSI | Six machine learning algorithms were used. The extreme gradient boosting model showed the best performance and was used to develop a web-based predictor tool. | (SSI) following lumbar spinal surgery | The extreme gradient boosting model achieved the highest predictive performance with an AUC of 0.923. |
| (OHNO et al., 2022) | Tokyo Medical University Hospital. | Japan | Retrospective cohort study | 730 patients who underwent surgery for stage II-III colon cancer between 2000 and 2018. | Model to predict SSIs based on immunological and nutritional markers trained on 730 prior patients | (SSIs), including superficial and deep incision infections. | The AI model achieved an AUC of 0.731 for predicting SSIs, comparable to conventional statistical models. |
| Rennert-May et al. (2022)  (Rennert-May et al., 2022) | Hospital Calgary, Alberta, Canada. | Canada | Retrospective cohort study | 3,536 cardiac implantable electronic device (CIED) procedures performed between January 1, 2013, and December 31, 2019. | The study used multiple approaches to identify SSIs, including three predefined administrative algorithms and two machine learning models. | Complex (SSIs) associated with CIEDs, such as deep infections or infections requiring device removal. | The machine learning model performed best with an AUC of 96.8%, while the best-performing traditional algorithm had an AUC of 94.6%. |
| (Scala et al., 2022) | “Federico II” University Hospital. | Italy | Retrospective cohort study | 4,031 patients who underwent surgery between 2015 and 2019, with 48 cases developing SSIs. | Logistic regression model for risk factors for SSIs based on EHRs data | SSIs, and includes infections occurring within 30 days post-surgery | The K-Nearest Neighbors (KNN) model showed the highest accuracy among the models tested, effectively predicting SSI risk despite the dataset's imbalance |
| (Zhu et al., 2022) | Multicenter study | China | Retrospective cohort study | Derivation Cohort: 3,982 immobile stroke patients (November 1, 2015, to June 30, 2016).  External Validation Cohort: 3,837 immobile stroke patients (November 1, 2016, to July 30, 2017). | Predictive models for UTI risk identification based on data | UTIs | The ensemble learning model achieved the best performance in identifying UTI risk, with an AUC of 82.2% in internal validation and 80.8% in external validation. Sensitivity was also high in both internal (80.9%) and external (81.1%) validation sets. |
| (Bonde et al., 2023) | Capital Region of Denmark, covering 11 hospitals. | Denmark | Retrospective observational study | Data from 389,865 surgical cases between January 2017 and October 2021, with over 4 million chart notes analyzed | Natural language processing model from EHR chart notes to identify postoperative SSIs trained on data | Superficial surgical site infections involving skin and subcutaneous tissue within 30 days of surgery. | The SAM model achieved an AUC of 0.989 for individual notes and 0.980 on a case level. It had a sensitivity of 0.604 and specificity of 0.996. |
| (Chen et al., 2023) | American College of Surgeons National Quality Improvement Program database | United States. | Retrospective observational study | 275,152 patients who underwent colorectal surgery from 2012 to 2019. | ML model for predicting of SSIs based on data | SSI, encompassing superficial, deep, and organ-space infections. | The deep neural network model showed a ROC AUC of 0.769, specificity of 50%, sensitivity 82% |
| (Flores-Balado et al., 2023) | Four public hospitals in Madrid. | Spain. | Retrospective observational study | 19,661 healthcare episodes involving hip replacement surgeries from the four hospitals. | Natural language processing and extreme gradient boosting model to predict SSIs, trained on EHR data | SSIs after hip replacement surgery. | The model had a sensitivity of 99.18% and specificity of 91.0% |
| (Huang et al., 2023) | The Third People's Hospital of Shenzhen | China | Pilot observational study | Personnel trained and evaluated by the system included 163 individuals before AITMS in preventing nosocomial infections) and 3,159 after its implementation. | Camera and speaker systems with integrated AI monitors for training on PPE and handwashing | HAIs | The accuracy of PPE donning/removal improved significantly with the AITMS, increasing from 52.15% to 98.14%, with a corresponding decrease in infection rate from 1.31% to 0.38% |
| (Jakobsen et al., 2023) | Hospitals in the North Denmark Region. | Denmark | Retrospective observational study | Data from 138,560 hospital admissions between January 1, 2017, and December 31, 2018. | ML model for UTIs prediction from EHR records at admission, trained on data from EHRs | UTIs | The best performing model was a deep neural network with an AUC of 0.758 on a full dataset; within 24 hours of admission the model was able to identify high-risk patients |
| (Kiser et al., 2023) | University of Utah Health system. | United States. | Retrospective observational study | Data from 9,185 surgical events between January 2016 and June 2021, with 434 cases of SSI (4.7%). | Long short-term memory model based on EHR data | SSIs | A maximum ROC AUC of 0.905 was achieved |
| Mamlook et al. (2023)  (Mamlook et al., 2023) | Data sourced from the American College of Surgeons’ National Surgical Quality Improvement Program | United States | Retrospective observational study | 2,882,526 surgical procedures from the database. | ML model for prediction of SSIs | SSIs | The DNN model achieved the highest predictive performance with an AUC of 0.8518, accuracy of 0.8518, precision of 0.8517, sensitivity of 0.8527 |
| (Rafaqat et al., 2023) | A tertiary care hospital. | Pakistan | Case-control study | Data from 113 patients with SSIs, with 62 cases of superficial SSIs (54.8%) and 51 cases of deep or organ space SSIs (45.2%). | ML model to predict timing and superficial or deep SSI | SSIs, specifically superficial vs. deep/organ space infections. | The XGBoost model achieved the highest predictive accuracy AUC-ROC of 0.84) for differentiating between superficial and deep/organ space SSIs. |
| (Sophonsri et al., 2023) | Hospital or healthcare setting focused on ventilated patients diagnosed HAP | United States | Retrospective observational study | ventilated patients diagnosed with HAP | ML model for risk factors for mortality based on data from 457 patients | HAP in patients on mechanical ventilation. | The model demonstrated reliable performance in predicting mortality risk in ventilated patients with HAP, showing potential for aiding clinical decisions and improving patient outcomes. |
| (Tsai et al., 2023) | Chi Mei Medical Center, an ED. | Taiwan | Retrospective observational study | 5,647 adult febrile ED patients who underwent blood cultures. | Random forest algorithm and logistic regression models to predict bacteraemia trained on 21 variables | Bacteraemia | The real-time prediction model can reduce unnecessary empirical antibiotic prescribing by up to 25% and was favourable compared to clinical risk scoring |
| (Wu et al., 2023) | Multiple acute care hospitals. | Canada. | Multicenter retrospective cohort study | 22,059 patients with 27,360 hospital admissions involving hip and knee arthroplasty from January 2013 to August 2020. | ML model for SSI detection trained on data from 22,059 patients | The optimal model (XGBoost using combined administrative and text data) achieved a ROC AUC of 0.906 and a PR AUC of 0.637, with an F1 score of 0.79. | The best model achieved ROC AUC of 0.906 and sensitivity 83.9% |
| (Bolton et al., 2024) | ICUs from multiple institutions | United States | Retrospective observational study | All patients admitted to ICU | ML model to predict when patients can switch from IV to oral antibiotics based on data from 10,362 ICU stays | Multiple HAIs | The AI model provided a fair and interpretable prediction system for determining when to switch from IV to oral antibiotics |
| (Cho et al., 2024) | Tertiary medical center. | Republic of Korea | Retrospective observational study | 1,652 Colon surgery cases from 2013 to 2014 | Five machine learning models were developed for SSIs | SSIs following colon surgery, specifically aiming to detect cases that would require intensive surveillance. | The deep neural network combined with a rule-based algorithm led to reducing the number of cases requiring chart review by 83.9% compared to conventional methods. The study concludes that ML can significantly improve the efficiency of SSI surveillance, |
| (Jakobsen et al., 2024) | North Denmark Regional Hospital \ Aalborg University Hospital in Denmark | Denmark | Retrospective cohort study | Information on 50 features from 138,250 admissions is included | The study used a Bayesian network model, integrating clinical expert knowledge and data-driven approaches to develop an interpretable model for early HA-UTI risk stratification. | UTIs | The model’s interpretability and use of expert knowledge make it suitable for clinical application, with the potential to support early intervention and infection prevention in hospitalized patients. |
| (Oonsivilai et al., 2018) | 100-bed children's hospital in North-West Cambodia between February 2013 and January 2016 | Cambodia | Retrospective observational study | 243 patients’ data with bloodstream infections were available for analysis | Multiple machine learning algorithms were developed and compared to predict antibiotic susceptibility, using clinical and microbiological data. | Various bacterial infections | A random forest model had an AUC of 0.80 for predicting susceptibility to antibiotics, including ceftriaxone, the results suggest that these models could potentially aid in clinical decision-making by guiding antibiotic prescription choices, contributing to improved management of antibiotic resistance. |
| (Lee et al., 2023) | Emergency department in a single tertiary university hospital. | South Korea. | retrospective study | 550 patients diagnosed with UTIs in the ED between January 2020 and June 2021. | The study used a GBDT model with SHAP for feature importance visualization | UTIs, with a focus on ciprofloxacin-resistant and ESBL-producing bacterial infections. | The GBDT model achieved an AUC of 0.829 for predicting ciprofloxacin resistance and 0.817 for ESBL positivity, the model could potentially guide ED clinicians in prescribing effective antibiotics, improving empirical therapy for UTIs and reducing unnecessary broad-spectrum antibiotic use |

**Appendix Table A1: *Characteristics of Included Individual Studies in the Umbrella Review***

AUC, area under the curve; CLABSI, central line-associated bloodstream infection; CVC, central venous catheter; EHR, electronic health record; ICU, intensive care unit; ML, machine learning; MRSA, meticillin-resistant *Staphylococcus aureus*; NPV, negative predictive value; PPE, personal protective equipment; PPV, positive predictive value; ROC, receiver operating characteristic; SSI, surgical site infection; UTI, urinary tract infection; SHAP, SHapley Additive exPlanations; ED, emergency department; ESBL, extended-spectrum beta-lactamase; DNN; deep neural network; HAP, Hospital Acquired Pneumonia;GBDT; Gradient-Boosted Decision Tree; AUROC, Area Under the Receiver Operating Characteristic

| Review Reference | Country | Publication Year | Study Design | Infection Types | AI Models Evaluated | Comparison Models (if any) | Main Performance Metrics | Key Findings |
| --- | --- | --- | --- | --- | --- | --- | --- | --- |
| (Scardoni et al., 2020) | Multiple countries | 2020 | Systematic review | Including CLABSI, SSI, CAUTI | Various models including Random Forest, Gradient Boosted Trees | Logistic regression, traditional scoring systems | Sensitivity, specificity, AUROC, PPV, NPV | AI models generally perform better than traditional methods in HAIs detection and prediction, though variability exists |
| (Radaelli et al., 2024) | Multiple countries | 2024 | Systematic review | SSIs, UTIs, and other various HAIs | Various models including Bayesian Networks, Random Forest, NLP, Neural Networks | Logistic regression, conventional scoring systems | AUROC, sensitivity, specificity, PPV, NPV | AI models demonstrated high sensitivity in predicting HAIs, with specific applications reducing workloads and HAIs incidence in hospitals. Key findings indicate that although AI models can improve infection surveillance and prevention, challenges such as high implementation costs and technological barriers remain. Proper implementation led to reductions in workload (up to 85%) and HAIs incidence in certain cases |
| (Baddal et al., 2024a) | Multiple countries | 2024 | Systematic review | CLABSI, CAUTI, VAP, CDI, SSI, and other HAIs | Various models including XGBoost, Random Forest, CNN, SVM, Gradient Boosted Trees, ANN | Logistic Regression, conventional scoring systems | AUROC, sensitivity, specificity, accuracy | AI models showed higher accuracy in predicting and diagnosing HAIs, with certain models excelling in specific infection types. |
| (Bomrah et al., 2024) | Multiple countries | 2024 | Scoping review | Sepsis | Various models including Random Forest, XG Boost, DNN | Traditional scoring systems, and occasionally clinician assessments | AUROC sensitivity, specificity, and other metrics depending on the study | Feature engineering significantly enhances predictive performance, with models like Random Forest and XG Boost showing strong results. Feature extraction and selection methods improve model sensitivity and AUROC, aiding in early and more accurate sepsis detection across diverse clinical settings​ |
| (Zhang et al., 2024) | Multiple countries | 2024 | Scoping review | VAP | Various models including Random Forest, XGBoost, Neural Networks, Decision Trees, Logistic Regression, K-Nearest Neighbors | None specified in document | AUC, Sensitivity, Specificity, Accuracy | AI models, especially ensemble learning methods like Random Forest and XGBoost, outperformed traditional models for VAP risk prediction. No studies used deep learning or large language models. Implementing and applying AI models in clinical settings still requires further research |

**Appendix Table A3**: *Characteristics of Included Scoping and Systematic Reviews in the Umbrella Review*

AUC, area under the curve; CLABSI, central line-associated bloodstream infection; CVC, central venous catheter; EHRs, electronic health records; ICU, intensive care unit; ML, machine learning; NPV, negative predictive value; PPV, positive predictive value; ROC, receiver operating characteristic; SSI, surgical site infection; UTI, urinary tract infection; SHAP, SHapley Additive exPlanations; ED, emergency department; ESBL, extended-spectrum beta-lactamase; DNN; deep neural network; HAP, Hospital Acquired Pneumonia; GBDT; Gradient-Boosted Decision Tree; AUROC, Area Under the Receiver Operating Characteristic; NLP;natural learning process, CAUTI; catheter-associated urinary tract infections, VAP;ventilator-associated pneumonia

Appendix A3: Individual studies classification into model development or implementation -evaluation studies

| Study (Author, Year) | Study Type | Target Users |
| --- | --- | --- |
| Rajeev Bopche et al., 2024 | Model Development | Infection control teams, Clinicians |
| Simioli et al., 2024 | Implementation & Evaluation | Healthcare personnel, Infection control teams |
| Lukasewicz Ferreira et al., 2024 | Implementation & Evaluation | Infection control professionals, Surveillance teams |
| Sohn et al., 2017 | Model Development | Surgical teams, Infection control specialists |
| Zachariah et al., 2020 | Model Development | Hospital clinicians, Infection control teams |
| Hopkins et al., 2020 | Model Development | Surgeons, Infection control teams |
| Kanjilal et al., 2020 | Model Development | Clinicians, Antibiotic stewardship programs |
| Barchitta et al., 2021 | Model Development | ICU clinicians, Infection control teams |
| dos Santos et al., 2021 | Implementation & Evaluation | Infection control professionals, Clinicians |
| Lind et al., 2021 | Model Development | Transplant teams, Clinicians |
| Møller et al., 2021 | Model Development | Hospital clinicians, Infection control teams |
| Petrosyan et al., 2021 | Model Development | Surgeons, Infection control teams |
| Walker et al., 2021 | Model Development | Pediatric clinicians, Infection control teams |
| Wang et al., 2021 | Model Development | Surgeons, Infection control teams |
| Caǧlayan et al., 2022 | Model Development | ICU clinicians, Infection control teams |
| Li et al., 2022 | Model Development | Clinicians, ICU teams |
| Liu et al., 2022 | Model Development | Surgeons, Infection control teams |
| OHNO et al., 2022 | Model Development | Colon surgeons, Infection control teams |
| Rennert-May et al., 2022 | Implementation & Evaluation | Cardiac implant teams, Infection control professionals |
| Scala et al., 2022 | Model Development | Surgeons, Infection control teams |
| Zhu et al., 2022 | Model Development | Stroke teams, Infection control |
| Bonde et al., 2023 | Model Development | Infection control professionals, Clinicians |
| Chen et al., 2023 | Model Development | Surgeons, Infection control teams |
| Flores-Balado et al., 2023 | Model Development | Surgeons, Infection control teams |
| Mamlook et al., 2023 | Model Development | Surgeons, Infection control teams |
| Huang et al., 2023 | Implementation & Evaluation | Healthcare personnel, Infection control teams |
| Jakobsen et al., 2023 | Model Development | Hospital clinicians, Infection control teams |
| Kiser et al., 2023 | Model Development | Surgeons, Infection control teams |
| Rafaqat et al., 2023 | Model Development | Surgeons, Infection control teams |
| Sophonsri et al., 2023 | Model Development | Clinicians, ICU teams |
| Tsai et al., 2023 | Implementation & Evaluation | Emergency department clinicians, Infection control teams |
| Oonsivilai et al., 2018 | Model Development | Clinicians, Antibiotic stewardship programs |
| Wu et al., 2023 | Model Development | Surgeons, Infection control teams |
| Cho et al., 2024 | Model Development | Surgeons, Infection control teams |
| Bolton et al., 2024 | Model Development | ICU clinicians, Antimicrobial stewardship programs |
| Jakobsen et al., 2024 | Model Development | Hospital clinicians, Infection control teams |
| Lee et al., 2023 | Model Development | ED clinicians |

**Appendix Table A4**: *Transparency and Reporting Quality of Included Individual Studies in the Umbrella Review*

| Study | Objectives | Source of Data | Participants | Predictors | Outcome Definition | Model Development and Validation | Model Performance and Interpretation |
| --- | --- | --- | --- | --- | --- | --- | --- |
| (Sohn et al., 2017) | Clear | Partially reported | Partially Reported | Well Reported | Clear | Adequate | Well Reported |
| Rajeev Bopche et al., 2024 | Clear | Adequately Reported | Partially Reported | Well Reported | Clear | Adequate | Well Reported |
| (Zachariah et al., 2020) | Clear | Adequately Reported | Partially Reported | Partially Reported | Clear | Adequate | Well Reported |
| (Hopkins et al., 2020) | Clear | Adequately Reported | Partially Reported | Partially Reported | Clear | Adequate | Well Reported |
| (Kanjilal et al., 2020) | Clear | Adequately Reported | Partially Reported | partially reported | Clear | Adequate | Well Reported |
| (Barchitta et al., 2021) | Clear | Adequately Reported | Partially Reported | Well Reported | Clear | Adequate | Well Reported |
| (dos Santos et al., 2021) | Clear | Adequately Reported | Partially Reported | Well Reported | Clear | Partially | Adequate |
| (Lind et al., 2021) | Clear | Adequately Reported | Partially Reported | Adequate | Clear | Adequate | Well Reported |
| (Møller et al., 2021) | Clear | Partially reported | Partially reported | Partially Reported | Clear | Adequate | Well Reported |
| (Petrosyan et al., 2021) | Clear | Adequately Reported | Partially Reported | Well Reported | Clear | Adequate | Well Reported |
| (Walker et al., 2021) | Clear | Adequately Reported | Partially Reported | Well Reported | Clear | Partially | Adequate |
| (Wang et al., 2021) | Clear | Adequately Reported | Well Reported | Well Reported | Clear | Adequate | Well Reported |
| (Caǧlayan et al., 2022) | Clear | Adequately Reported | Partially Reported | Well Reported | Clear | Adequate | Well Reported |
| (Li et al., 2022) | Clear | Adequately Reported | Partially Reported | Well Reported | Clear | Adequate | Well Reported |
| (Liu et al., 2022) | Clear | Adequately Reported | Partially Reported | Well Reported | Clear | Adequate | Well Reported |
| (OHNO et al., 2022) | Clear | Adequately Reported | Partially Reported | Well Reported | Clear | Partially | Well Reported |
| (Rennert-May et al., 2022) | Clear | Adequately Reported | Well Reported | Partially | Clear | Partially | Well Reported |
| (Scala et al., 2022) | Clear | Adequately Reported | Well Reported | Well Reported | Clear | Partially | Well Reported |
| (Zhu et al., 2022) | Clear | Adequately Reported | Well Reported | Well Reported | Clear | Adequate | Well Reported |
| (Bonde et al., 2023) | Clear | Adequately Reported | Partially Reported | Well Reported | Clear | Adequate | Well Reported |
| (Chen et al., 2023) | Clear | Adequately Reported | Well Reported | Well Reported | Clear | Adequate | Well Reported |
| (Flores-Balado et al., 2023) | Clear | Adequately Reported | Well Reported | Well Reported | Clear | Adequate | Well Reported |
| (Mamlook et al., 2023) | Clear | Adequately Reported | Partially Reported | Well Reported | Clear | Adequate | Well Reported |
| (Huang et al., 2023) | Clear | Adequately Reported | Well Reported | Well Reported | Clear | Partially | Well Reported |
| (Jakobsen et al., 2023) | Clear | Adequately Reported | Well Reported | Well Reported | Clear | Adequate | Well Reported |
| (Kiser et al., 2023) | Clear | Adequately Reported | Partially Reported | Well Reported | Clear | Adequate | Well Reported |
| (Rafaqat et al., 2023) | Clear | Adequately Reported | Partially Reported | Well Reported | Clear | Adequate | Well Reported |
| (Sophonsri et al., 2023) | Clear | Adequately Reported | Partially Reported | Well Reported | Clear | Adequate | Well Reported |
| (Tsai et al., 2023) | Clear | Adequately Reported | Well Reported | Well Reported | Clear | Adequate | Well Reported |
| (Oonsivilai et al., 2018) | Clear | Adequately Reported | Partially Reported | Well Reported | Clear | Adequate | Well Reported |
| (Wu et al., 2023) | Clear | Adequately Reported | Partially Reported | Well Reported | Clear | Adequate | Well Reported |
| (Cho et al., 2024) | Clear | Adequately Reported | Partially Reported | Well Reported | Clear | Adequate | Well Reported |
| (Bolton et al., 2024) | Clear | Adequately Reported | Partially Reported | Well Reported | Clear | Adequate | Well Reported |
| (Jakobsen et al., 2024) | Clear | Adequately Reported | Partially Reported | Well Reported | Clear | Adequate | Well Reported |
| (Lee et al., 2023) | Clear | Adequately Reported | Partially Reported | Well Reported | Clear | Adequate | Well Reported |
| (Lukasewicz Ferreira et al., 2024a) | Clear | Adequately Reported | Well Reported | Well Reported | Clear | Adequate | Well Reported |
| (Simioli et al., 2024) | Clear | Adequately Reported | Partially Reported | Well Reported | Clear | Adequate | Well Reported |

**Appendix Table A5**: *AMSTAR 2 Quality Assessment* (Scardoni et al., 2020)

| (Scardoni et al., 2020) | Description | Rating | Comments |
| --- | --- | --- | --- |
| 1. PICO Components | Research questions and inclusion criteria include PICO components (Population, Intervention, Comparison, Outcome). | Partial Yes | Clearly defines population, intervention, comparison, and outcome with no direct user of PICO directly. |
| 2. Protocol Registration | Protocol registered before starting the review (e.g., on PROSPERO). | No | No mention of pre-registration. |
| 3. Explanation of Study Design | Clear explanation of the study designs selected for inclusion. | Yes | Justified selection of retrospective/prospective cohorts and RCTs. |
| 4. Comprehensive Literature Search | Comprehensive search strategy across multiple databases and sources. | Partial Yes | Databases were searched, but keyword and search limit details are limited. |
| 5. Study Selection in Duplicate | Two independent reviewers screened studies. | Yes | Screening done independently by two authors. |
| 6. Data Extraction in Duplicate | Data extracted by two independent reviewers. | Yes | Two authors conducted data extraction. |
| 7. Excluded Studies | List and justification of excluded studies provided. | No | No list of excluded studies with reasons. |
| 8. Description of Included Studies | Detailed description of each study's characteristics. | Yes | Includes infection type, sample size, AI model, outcomes. |
| 9. Risk of Bias Assessment | Appropriate risk of bias tool used for each study type. | Yes | Used NOS for observational studies and Cochrane Risk of Bias for RCTs. |
| 10. Funding Sources for Studies | Reporting of funding sources or conflicts of interest in included studies. | Partial Yes | Some mention of private sector involvement. |
| 11. Appropriate Meta-analysis Methods | Use of appropriate methods for any meta-analysis. | Yes | Pooling methods were outlined if appropriate. |
| 12. Risk of Bias Impact | Assessment of risk of bias impact on the review results. | No | No specific analysis of bias impact on synthesis results. |
| 13. Bias Consideration in Discussion | Consideration of risk of bias when interpreting results. | Partial Yes | Heterogeneity noted, but not linked to individual study biases. |
| 14. Explanation of Heterogeneity | Explanation of heterogeneity in the results. | Yes | Variability discussed based on infection type, AI models, settings. |
| 15. Publication Bias | Investigation of publication bias in synthesis. | No | Publication bias not addressed. |
| 16. Conflict of Interest for Review | No conflict of interest declared | | |

**Appendix Table A6**: *AMSTAR 2 Quality Assessment* (Radaelli et al., 2024)

| (Radaelli et al., 2024) | Description | Rating | Comments |
| --- | --- | --- | --- |
| 1. PICO Components | Research questions and inclusion criteria include PICO components (Population, Intervention, Comparison, Outcome). | Partial Yes | Clearly defines population, intervention, comparison, and outcome. |
| 2. Protocol Registration | Protocol registered before starting the review (e.g., on PROSPERO). | No | No mention of pre-registration. |
| 3. Explanation of Study Design | Clear explanation of the study designs selected for inclusion. | Yes | Justified selection of retrospective/prospective cohorts and RCTs. |
| 4. Comprehensive Literature Search | Comprehensive search strategy across multiple databases and sources. | Partial Yes | Databases were searched, but keyword and search limit details are limited. |
| 5. Study Selection in Duplicate | Two independent reviewers screened studies. | Yes | Screening done independently by two authors. |
| 6. Data Extraction in Duplicate | Data extracted by two independent reviewers. | Yes | Two authors conducted data extraction. |
| 7. Excluded Studies | List and justification of excluded studies provided. | No | No list of excluded studies with reasons. |
| 8. Description of Included Studies | Detailed description of each study's characteristics. | Yes | Includes infection type, sample size, AI model, outcomes. |
| 9. Risk of Bias Assessment | Appropriate risk of bias tool used for each study type. | Partial Yes | Used NOS for observational studies and Cochrane Risk of Bias for RCTs. |
| 10. Funding Sources for Studies | Reporting of funding sources or conflicts of interest in included studies. | Partial Yes | Some mention of private sector involvement. |
| 11. Appropriate Meta-analysis Methods | Use of appropriate methods for any meta-analysis. | Yes | Pooling methods were outlined if appropriate. |
| 12. Risk of Bias Impact | Assessment of risk of bias impact on the review results. | No | No specific analysis of bias impact on synthesis results. |
| 13. Bias Consideration in Discussion | Consideration of risk of bias when interpreting results. | Partial Yes | Heterogeneity noted, but not linked to individual study biases. |
| 14. Explanation of Heterogeneity | Explanation of heterogeneity in the results. | Yes | Variability discussed based on infection type, AI models, settings. |
| 15. Publication Bias | Investigation of publication bias in synthesis. | No | Publication bias not addressed. |
| 16. Conflict of Interest for Review | No conflict of interest declared | | |

**Appendix Table A7**: *AMSTAR 2 Quality Assessment* (Baddal et al., 2024b)

| (Baddal et al., 2024b) | Description | Rating | Comments |
| --- | --- | --- | --- |
| 1. PICO Components | Research questions and inclusion criteria include PICO components (Population, Intervention, Comparison, Outcome). | Yes | The review clearly defines the population (hospital settings), intervention (AI for HAI diagnosis/prevention), and outcomes (detection and prevention effectiveness). |
| 2. Protocol Registration | Protocol registered before starting the review (e.g., on PROSPERO). | No | No indication of protocol registration is provided in the document. |
| 3. Explanation of Study Design | Clear explanation of the study designs selected for inclusion. | Yes | The review justifies the inclusion of various study designs relevant to AI applications in HAI prevention. |
| 4. Comprehensive Literature Search | Comprehensive search strategy across multiple databases and sources. | Partial Yes | The review includes a search across databases; however, specific keywords or search details are not fully disclosed. |
| 5. Study Selection in Duplicate | Two independent reviewers screened studies. | Yes | Study selection was conducted by two reviewers, enhancing reliability. |
| 6. Data Extraction in Duplicate | Data extracted by two independent reviewers. | Yes | Data extraction was performed independently by two authors. |
| 7. Excluded Studies | List and justification of excluded studies provided. | No | The review does not provide a list of excluded studies or reasons for exclusion. |
| 8. Description of Included Studies | Detailed description of each study's characteristics. | Yes | Provides study characteristics, including infection types, AI models, outcomes, and settings. |
| 9. Risk of Bias Assessment | Appropriate risk of bias tool used for each study type. | Partial Yes | Risk of bias is assessed, but details on the specific tool used are limited. |
| 10. Funding Sources for Studies | Reporting of funding sources or conflicts of interest in included studies. | Partial Yes | Mentions funding sources generally, but this is not applied consistently to all studies. |
| 11. Appropriate Meta-analysis Methods | Use of appropriate methods for any meta-analysis. | Yes | No meta-analysis conducted due to heterogeneity, which is appropriate for the diversity in study designs and AI models. |
| 12. Risk of Bias Impact | Assessment of risk of bias impact on the review results. | No | There is no specific discussion of how bias might affect the synthesis of results. |
| 13. Bias Consideration in Discussion | Consideration of risk of bias when interpreting results. | Partial Yes | Discusses heterogeneity but lacks explicit linkage to study-level biases in the interpretation. |
| 14. Explanation of Heterogeneity | Explanation of heterogeneity in the results. | Yes | Heterogeneity is addressed, considering the variety of AI models, infection types, and healthcare settings. |
| 15. Publication Bias | Investigation of publication bias in synthesis. | No | Publication bias is not assessed in this review. |
| 16. Conflict of Interest for Review | No conflict of interest declared | | |

**Appendix Table A8**: *AMSTAR 2 Quality Assessment* (Bomrah et al., 2024)

| (Bomrah et al., 2024) | Description | Rating | Comments |
| --- | --- | --- | --- |
| 1. PICO Components | Research questions and inclusion criteria include PICO components (Population, Intervention, Comparison, Outcome). | No | Clearly defines population (ICU/ED patients), intervention (ML models), with some detail on outcomes.  Scoping review (PRISMA Scoping reviews |
| 2. Protocol Registration | Protocol registered before starting the review (e.g., on PROSPERO). | No | No indication of protocol pre-registration. |
| 3. Explanation of Study Design | Clear explanation of the study designs selected for inclusion. | Yes | Justified inclusion of retrospective analyses and various ML models for sepsis prediction. |
| 4. Comprehensive Literature Search | Comprehensive search strategy across multiple databases and sources. | Yes | Literature search covered databases like PubMed, Embase, and Scopus, with a focus on recent studies. |
| 5. Study Selection in Duplicate | Two independent reviewers screened studies. | No | No explicit mention of study selection by independent reviewers. |
| 6. Data Extraction in Duplicate | Data extracted by two independent reviewers. | Yes | Data extraction was performed and validated by additional reviewers, ensuring accuracy. |
| 7. Excluded Studies | List and justification of excluded studies provided. | No | No detailed list or justification for excluded studies. |
| 8. Description of Included Studies | Detailed description of each study's characteristics. | Yes | Includes details such as infection type, ML model used, and main performance metrics. |
| 9. Risk of Bias Assessment | Appropriate risk of bias tool used for each study type. | No | No formal risk of bias assessment for included studies. |
| 10. Funding Sources for Studies | Reporting of funding sources or conflicts of interest in included studies. | No | No information on funding sources for the included studies. |
| 11. Appropriate Meta-analysis Methods | Use of appropriate methods for any meta-analysis. | Not Applicable | Meta-analysis not conducted as this is a scoping review. |
| 12. Risk of Bias Impact | Assessment of risk of bias impact on the review results. | No | No discussion of how bias in the studies might affect findings. |
| 13. Bias Consideration in Discussion | Consideration of risk of bias when interpreting results. | No | Discussion lacks consideration of bias from individual studies. |
| 14. Explanation of Heterogeneity | Explanation of heterogeneity in the results. | Yes | Variability discussed based on infection type, ML models, and feature engineering methods. |
| 15. Publication Bias | Investigation of publication bias in synthesis. | No | Publication bias not addressed. |
| 16. Conflict of Interest for Review | No conflict of interest declared | | |

**Appendix Table A9**: *AMSTAR 2 Quality Assessment* (Zhang et al., 2024)

| (Zhang et al., 2024) | Description | Rating | Comments |
| --- | --- | --- | --- |
| 1. PICO Components | Research questions and inclusion criteria include PICO components (Population, Intervention, Comparison, Outcome). | No | Clearly defines the population (ICU patients), intervention (AI models), with details on predictors and outcomes. Scoping review (PRISMA Scoping reviews |
| 2. Protocol Registration | Protocol registered before starting the review (e.g., on PROSPERO). | No | No indication of a registered protocol. |
| 3. Explanation of Study Design | Clear explanation of the study designs selected for inclusion. | Yes | Provides a clear rationale for including retrospective and prospective studies in ICU settings. |
| 4. Comprehensive Literature Search | Comprehensive search strategy across multiple databases and sources. | Yes | Conducted a comprehensive search in multiple databases, including PubMed, Cochrane, and Embase. |
| 5. Study Selection in Duplicate | Two independent reviewers screened studies. | Yes | Screening was done independently by two reviewers to minimize bias. |
| 6. Data Extraction in Duplicate | Data extracted by two independent reviewers. | Yes | Data extraction was independently conducted by two reviewers. |
| 7. Excluded Studies | List and justification of excluded studies provided. | No | No list or rationale for excluded studies was included in the review. |
| 8. Description of Included Studies | Detailed description of each study's characteristics. | Yes | Provides details on study design, country, sample size, and AI model characteristics for each included study. |
| 9. Risk of Bias Assessment | Appropriate risk of bias tool used for each study type. | No | No formal risk of bias assessment was conducted for included studies. |
| 10. Funding Sources for Studies | Reporting of funding sources or conflicts of interest in included studies. | No | No information on funding sources for the included studies. |
| 11. Appropriate Meta-analysis Methods | Use of appropriate methods for any meta-analysis. | Not Applicable | Meta-analysis was not performed as this is a scoping review. |
| 12. Risk of Bias Impact | Assessment of risk of bias impact on the review results. | No | No discussion on how biases in studies might impact the review’s conclusions. |
| 13. Bias Consideration in Discussion | Consideration of risk of bias when interpreting results. | No | No explicit discussion of bias in the review's interpretation of results. |
| 14. Explanation of Heterogeneity | Explanation of heterogeneity in the results. | Yes | Discusses variability in AI models, data types, and clinical settings, highlighting their impact on study outcomes. |
| 15. Publication Bias | Investigation of publication bias in synthesis. | No | Publication bias was not addressed in the review. |
| 16. Conflict of Interest for Review | No conflict of interest declared | | |
